# Chicago Medical Society

**Published:** 1869-08-15

**Authors:** Hiram Wanzer

**Affiliations:** Secretary


### SOCIETY REPORTS.

---

### *Chicago Medical Society.*

FRIDAY EVENING, July 9, 1869.

Regular meeting Chicago Medical Society, President Bogue in the chair. The election of new members being in order, Drs. Wm. A. Barstow, H. Hooper, H. Hurd and Charles Bendike were unanimously elected.

Report of Committees: none being ready, the meeting then proceeded with the discussion: "Is Hydrophobia amenable to treatment?" Dr. Paoli opened by relating the interest it had, not only to people but to the profession itself. The disease, although it had been much observed, was still in obscurity, yet every effort was made to treat it conscientiously. The death of Mr. Goodwille, from hydrophobia, had been taken up by all the Chicago papers and highly colored modes of treatment were admitted by them. The New York *Medical Gazette* had reprinted the report from the Chicago papers and commented thereon freely, speaking of hydrophobia as incurahle, etc., and the treatment adopted as inhuman. Dr. Paoli took great pains to examine into the case and found that it was partly right and wrong. The steam-bath was used in Goodwille's case up to 135 deg., but hot water had not been used. Mr. Goodwille, who was very fond of dogs, picked up the one that bit him, whom he suspected of being mad, but did not notice any effects until two months afterwards. It was not true that the patient was immersed by force. It was also untrue that there was any barking sound from his throat. He was rational the whole time, and when he felt a convulsion coming on he warned his family to keep away from him. It was necessary to learn of the dog's disease first. That hydrophobia is very rare was shown by statistics, there being only 25 deaths in 36,000,000 of people. It would be easy to study the case if all who were reported to have died of it were, really, the victims of that disease. If it was more recognizable in dogs there was little doubt of the suppression of the disease. The first symptom in the dog was its sullen and melancholy appearance, and its illusive biting at imaginary objects. In the dog there were similar smyptoms to those exhibited in man. As the symptoms increased the dog appeared thirsty, but did not drink, only snapping at the water. He was in a paralytic state. In the human being there was no snapping, yet he was in a melancholy state before the symptoms commenced. The skin was very sensitive, and a slight draught would cause convulsions. In the dog there was precisely an opposite symptom. The man was rational, the dog was furious. The man died asphyxiated, the dog paralytic. It would be interesting to examine the subject and mention the various modes of treatment. It must be admitted there was no remedy, but there were preventives. The absorption of this poison could be stopped. Constitution has something to do with taking the disease, and a great many that were bitten by dogs that were mad recovered. Amputation was recommended, but that was barbarous. He suggested the application of the hot iron. A Russian physician discovered, many years ago, under the tongue of a victim, a small vescicle, and said if this could be removed the patient would recover. This he doubted partially. The physician qualified his statement by saying if, before the 22d day, these vescicles could be removed there was no fear of fatal consequences. There had not been one remedy, external or internal, successful. Bleeding, mercury, arsenic, iron, gold, bromine, silver, and sulphur preparations had been tried, and all failed. The leaves of the Guaco of South America had proved unavailing. A French physician suggested cutting out the wound. If remedies could be immediately applied the patient could be saved. A turnquet had been used, but it only delayed death. Opium and chloroform had been employed. Beneficial effect can then only be expected when the public is made to know the symptoms of rabies in a dog.

Dr. Smith thought, though the question was the treatment of the disease, yet it was necessary to gain some knowledge of its origin. Many physicians, himself included, thought it was only a modified form of tetanus, and not a disease of special contagion. He had made inoculution from the saliva from the mouth of a mad dog, on seven dogs, a horse and a cow, and no results followed, as they invariably did in all cases of contagion, as small pox, syphilis, and others. He had seen a case of furious tetanus produced by an almost imperceptible wound by a pair of scissors falling on the patient's toe, the symptoms coming only on the seventh day. The man refused water and died with all the symptoms of what was called hydrophobia. He thought special observations should be made. He had lived in the city for fourteen years, and had been called to attend diseases similar to hydrophobia, and found them not that disease, and never had seen one case. Barking was a symptom which occurred in inflammation of the cerebellum. He recently attended an epileptic patient who barked like a dog. If that occurred in chorea, why not in any other form of inflammation of the cerebellum. He believed that a bite by a diseased dog might cause something more than tetanus, and did not like to be understood that hydrophobia was nothing more than tetanus, but that nothing special was attached to it, and, in his opinion, it was not a special disease. He had had an interesting case a year ago, in the family of a physician in the city, where a lady had been bitten by a dog. On inspection, the dog certainly evinced symptoms of hydrophobia, and died the next evening. He had found, in his post mortem examination, every symptom generally given by doctors. The lady, however, had exhibited no symptoms, and was now living and in general health. She was treated with bromide of potassium, and nothing resulted. He objected exceedingly to the poisoning of dogs on the streets, which demoralized the police and the children who saw them die; and was it safe to put in the hands of policemen, political appointees, means enough to poison two or three thousand people? The health of the city was injured by having dead dogs about it, and they were at liberty to die anywhere, and frequently died in annoying places He had seen malignant carbuncle spread by the flies and insects that preyed on these dead animals. If dogs must be killed, other ways and means might be employed, some of which he recommended.

Dr. Danforth said the idea of the vescicle was exploded. If one was there it had no connection with the disease.

Dr. Loverin said the disease had received its name from one of its symptoms, a fear of water. The dog, fox and wolf were capable of producing the disease, and it was popularly believed that they inoculated victims by their saliva. Hydrophobia had many symptoms in common with tetanus, but the fear of water was peculiar to itself. In tetanus we have no fear of water. A prophylactic measure immediately after the bite, was incision of the wound, but the question arose, how long after the bite would the excision be useful? Many eminent men believed that excision before suppuration took place was safe, and was to be recommended. After the symptoms were well represented no known method could restore the patient. He had never seen a case of hydrophobia, but believed there was such a disease.

The symptoms of madness in dogs had been described very well by the gentleman who first spoke. When a dog was rabid he had a musical howl.

Dr. Davis considered the symptoms purely nervous, resulting from direct irritation of the nerve and spinal centres, medulla oblongata, etc. Cases of hydrophobia are very rare, and literature is made up of illy-reported newspaper items, and very unreliable. A writer may talk about it and may never nave seen a case. Medicines must be sedative, and no heroic treatment necessary, and he would never advocate remedies which would kill a well person. Similar causes we meet with of an irritating nature to the nerve-centres, controlling respiration, circulation, in menigitis, cerebrospinalis epidemica. No harsh means in the treatment should ever be employed. Belladonna and bromides, alternately, with full doses of soda sulphites. Permanganate of potassa to counteract animal poison may be given, as all cases prove fatal any how, but there was an error in trying every thing. and nothing effectual, The dog must be diagnosed to truly have the disease before incision is practiced, but where it results in mutilation no one would feel justified in attempting it.

On motion, society adjourned.

HIRAM WANZER, *Secretary.*

